# Pleural fluid MYD88 L265P mutation supporting diagnosis and decision to treat extramedullary Waldenstrom’s macroglobulinemia: a case report

**DOI:** 10.1186/s13256-020-02404-x

**Published:** 2020-07-13

**Authors:** Martin Barnes, Pritha Sharma, Vikas Kumar, Alan Kaell, William LiPera

**Affiliations:** 1 Department of Internal Medicine, Mather Hospital/Northwell Health, 75 North Country Rd, Port Jefferson, NY 11777 USA; 2 New York Cancer & Blood Specialists, 49 Route 347, Port Jefferson Station, NY 11776 USA

**Keywords:** Extramedullary, Waldenstrom’s macroglobulinemia, Lymphoplasmacytic, Allele-specific polymerase chain reaction (AS-PCR), MYD88 L265P Mutation, Pleural fluid, Pulmonary, Thoracentesis, Ibrutinib

## Abstract

**Background:**

Our case of a patient with untreated lymphoplasmacytic lymphoma/Waldenstrom’s macroglobulinemia with extramedullary pleural effusion is the first documented case of pleural fluid MYD88 L265P mutation status in a community hospital setting. Our patient was intolerant to 420 mg ibrutinib, but still achieved a lasting complete remission, as defined by National Comprehensive Cancer Network guidelines, with a dose reduction to 240 mg of ibrutinib.

**Case presentation:**

A 72-year-old Caucasian (white) man diagnosed with monoclonal immunoglobin M kappa lymphoplasmacytic lymphoma/Waldenstrom’s macroglobulinemia monitored without treatment for 2 years, presented with dyspnea and a left pleural effusion. At presentation, computed tomography scans of his chest, abdomen, and pelvis showed layering left pleural effusion and para-aortic lymphadenopathy. Pleural fluid cytology demonstrated B-cell lymphoma of the lymphoplasmacytic subtype, with monoclonal kappa B-cell population on flow and a positive MYD88 L265P mutation. The pleural effusion recurred post-thoracentesis and he achieved a lasting complete remission as defined by National Comprehensive Cancer Network guideline with 240 mg ibrutinib.

**Conclusions:**

Our discussion details a comprehensive literature review of extramedullary pulmonary involvement in Waldenstrom’s macroglobulinemia. Establishing a malignant etiology for pleural effusion in Waldenstrom’s macroglobulinemia can be challenging, as standard techniques may be insensitive. Allele-specific polymerase chain reaction for detecting MYD88 L265P mutations is more sensitive for confirming lymphoplasmacytic lymphoma/Waldenstrom’s macroglobulinemia in pleural fluid. Extramedullary pulmonary involvement usually presents post-diagnosis of Waldenstrom’s macroglobulinemia and responds well to Waldenstrom’s macroglobulinemia-directed treatment regimens. Allele-specific polymerase chain reaction is a sensitive assay for detecting MYD88 L265P mutations in pleural fluid to support the diagnosis of malignant pleural effusion in the setting of Waldenstrom’s macroglobulinemia and helps guide the treatment decision to use ibrutinib. Although intolerant of ibrutinib 420 mg, our patient achieved complete and sustained remission of pleural effusion with a dose of 240 mg with progression free survival of over 30 months.

## Background

Extramedullary (EM) pulmonary involvement occurs in < 2% of patients with Waldenstrom’s Macroglobulinemia (WM)/lymphoplasmacytic lymphoma (LPL). Manifestations include unilateral pleural effusion; most malignant effusions occur after treatment for WM [1]. A literature review of the diagnostic usefulness of pleural fluid MYD88 L265P mutation status showed it was restricted to academic, tertiary-care centers [2]. Our case of a patient with untreated WM/LPL with EM pleural effusion is the first documented case with pleural fluid MYD88 L265P mutation status in a community hospital setting. The effusion resolved completely despite tolerating only one-half of the recommended dose of ibrutinib therapy [3].

## Case presentation

A 72-year-old Caucasian (white) man with a history of diffuse large B-cell lymphoma (DLBCL), immunoglobin M (IgM) monoclonal gammopathy of undetermined significance (MGUS), and chronic back pain secondary to osteoarthritis presented with dyspnea and a left pleural effusion. His social history was notable for a 60 pack-year cigarette smoking, and he had cut down to five cigarettes a day after diagnosis of non-Hodgkin’s lymphoma (NHL). He denied any significant past alcohol abuse, illicit drug abuse, or prescription drug abuse and reported consuming a beer or two on rare social occasions. Our patient was adopted so he is unaware of any past family history in regard to his parents. His brother has high blood pressure; otherwise he reported no other known malignancy or cardiopulmonary disease in the family. He worked as a mechanic and retired 20 years ago. He denied any known environmental exposures or allergies. Home medications included a daily probiotic, florastor 250 mg 2 tablets once daily, and acetaminophen/oxycodone – 325 mg/5 tablets once a day as needed for severe back pain. Our patient explained that he took opiate pain medication two to three times a week. Seven years prior, a bone marrow (BM) biopsy had revealed DLBCL, stage IVB, and he achieved complete remission (CR) with rituximab, cyclophosphamide, doxorubicin, vincristine, prednisolone (R-CHOP). Two years prior, he was diagnosed with monoclonal IgM kappa MGUS and BM biopsy revealed B-cell lymphoma with plasmacytic differentiation (10% of the marrow cellularity). The differential diagnosis at this time included LPL and marginal zone lymphoma. Given that he was asymptomatic at this time, he was monitored without treatment.

**Fig. 1:**
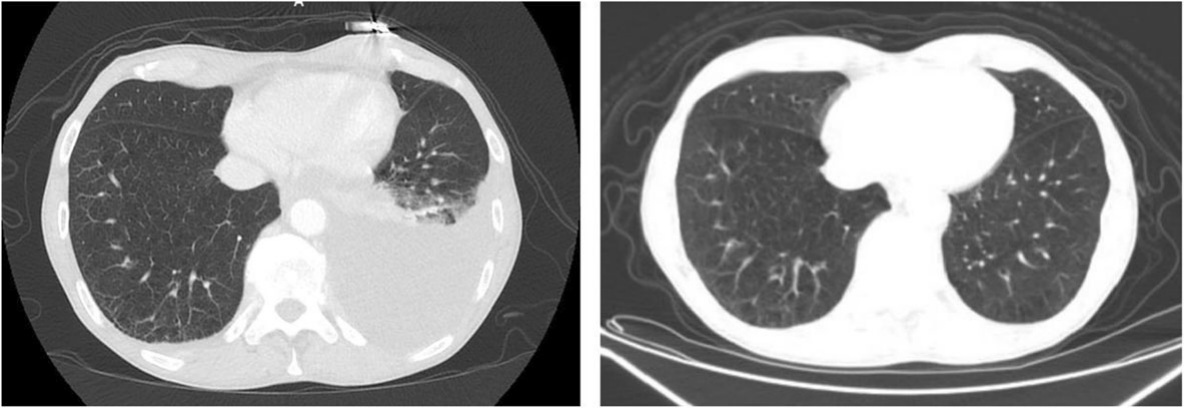
Chest CT demonstrated left-sided pleural effusion on initial presentation and complete resolution status post ibrutinib treatment on follow-up chest CT imaging. *CT* computed tomography, *PF* pleural fluid

In the Emergency Department (ED), his vital signs were a pulse of 88 beats per minute, his blood pressure was 145/62, and temperature was 98.7 degrees Fahrenheit. His oxygen saturation was 95% on room air and he was mildly tachypneic with a respiratory rate of 24 breaths per minute. At physical examination, he appeared well, he was well nourished, awake, and in no apparent distress. An ear, nose, mouth and throat examination showed clear nasal mucosa and normal mouth mucosa. His head was atraumatic/normocephalic. His heart rate was normal, with a regular rhythm, heart sounds were S1, S2, and there were no murmurs, rubs or gallops. There were rales in his left lung base without wheeze or rhonchi. His abdomen was soft, non-distended, and non-tender. His lumbar spine showed a mild reduction in range of motion. He showed superficial bilateral inguinal lymphadenopathy. He was alert and oriented × 4, with no focal deficits, and no motor or sensory deficits. His CN II-XII were intact, extremities showed no cyanosis or edema, and his skin was dry, intact, with no rash. Our patient was given one ipratropium bromide/albuterol nebulizer treatment and two doses of morphine 1 mg for back pain. During the admission, our patient received tramadol 50 mg once for back pain and required no other medication. His complete blood count showed a white blood cell count of 6.2 × 109/L, hemoglobin of 13.8 g/dL, platelet count of 156 × 109/L with normal differential. The complete metabolic panel showed sodium of 141 mEq/L, potassium of 3.8 mEq/L, blood urea nitrogen of 18 mg/dL and creatinine of 1.0 mg/dL, glucose of 95 mg/dL, calcium of 9.2, albumin 3.8 g/dL, aspartate aminotransferase of 23 IU/L, and alanine aminotransferase of 32 IU/L, alkaline phosphatase of 46, and total bilirubin of 0.6. The urinalysis was within normal limits with no evidence of hematuria, pyuria, nitrates were not detected, and there were no bacteria. A computed tomography scan of his chest (Fig. 1), abdomen, and pelvis showed layering left pleural effusion and para-aortic retroperitoneal lymphadenopathy extending to the iliacs and superficial inguinal nodes. Pleural fluid (PF) cytology was positive for malignant cells consistent with B-cell lymphoma and positive for MYD88 L265P mutation.

**Fig. 2:**
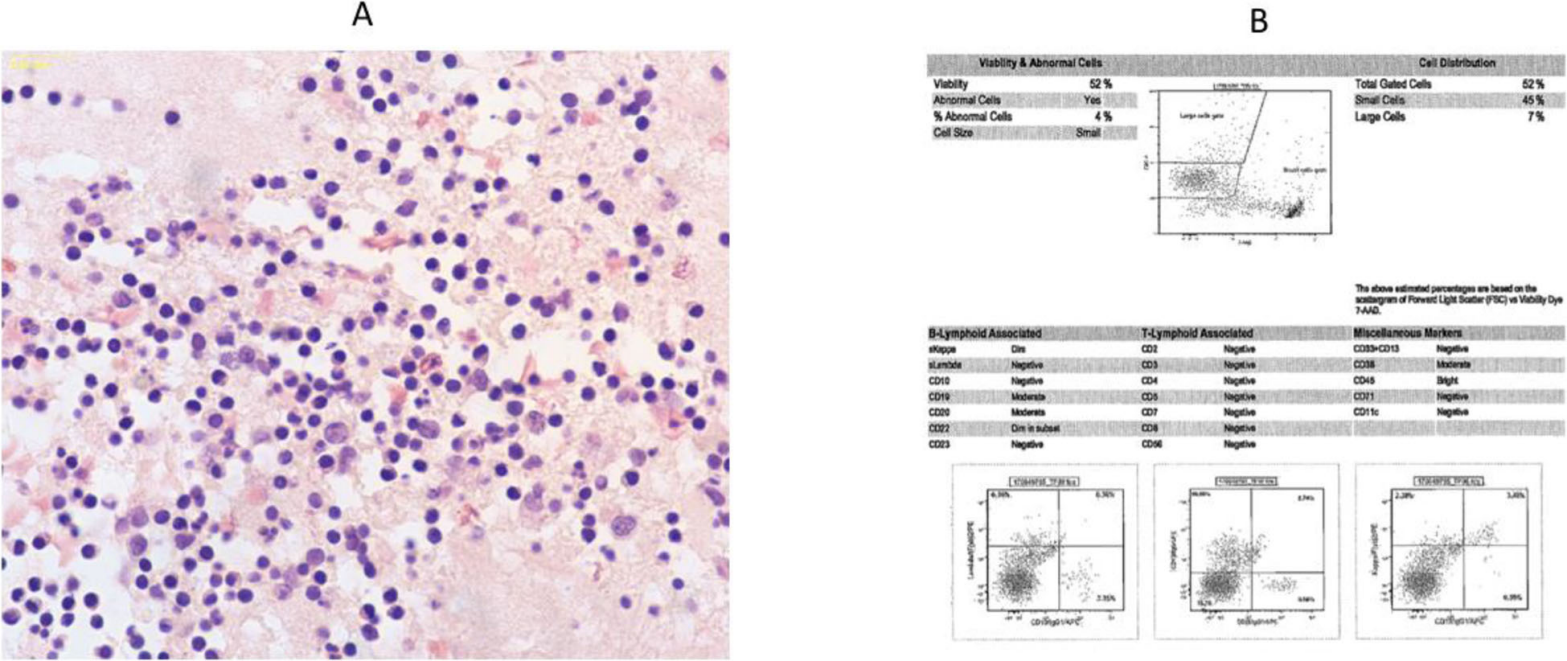
(**a**) PF cytology shows mainly small lymphocytes, positive for MYD88 mutation. (**b**) Flow cytometry shows monoclonal kappa B-cell population consistent with B-cell lymphoma. *LPL/WM* lymphoplasmacytic lymphoma/Waldenstrom’s macroglobulinemia, *PF* pleural fluid

PF flow cytometry showed monoclonal kappa B-cell population with moderate CD19, CD20, CD22, and CD38 expression (Fig. 2).

**Fig. 3:**
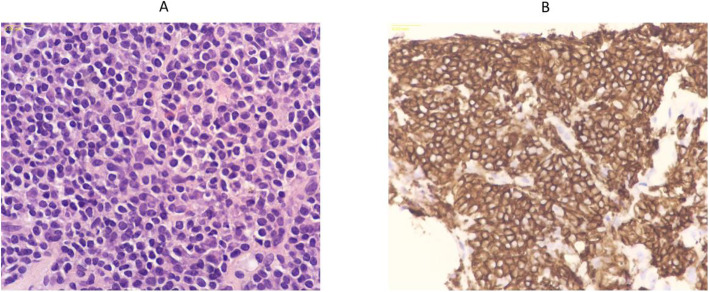
Inguinal lymph node biopsy showing (**a**) small lymphocytes and scattered plasmacytoid cells (**b**) Immunohistochemistry showing CD20 positive stain

Inguinal lymph node biopsy showed lymphoplasmacytic, monoclonal B-cell population, CD20-positive, CD5-negative, CD10-negative B-cells, consistent with LPL/WM (Fig. 3).

**Fig. 4:**
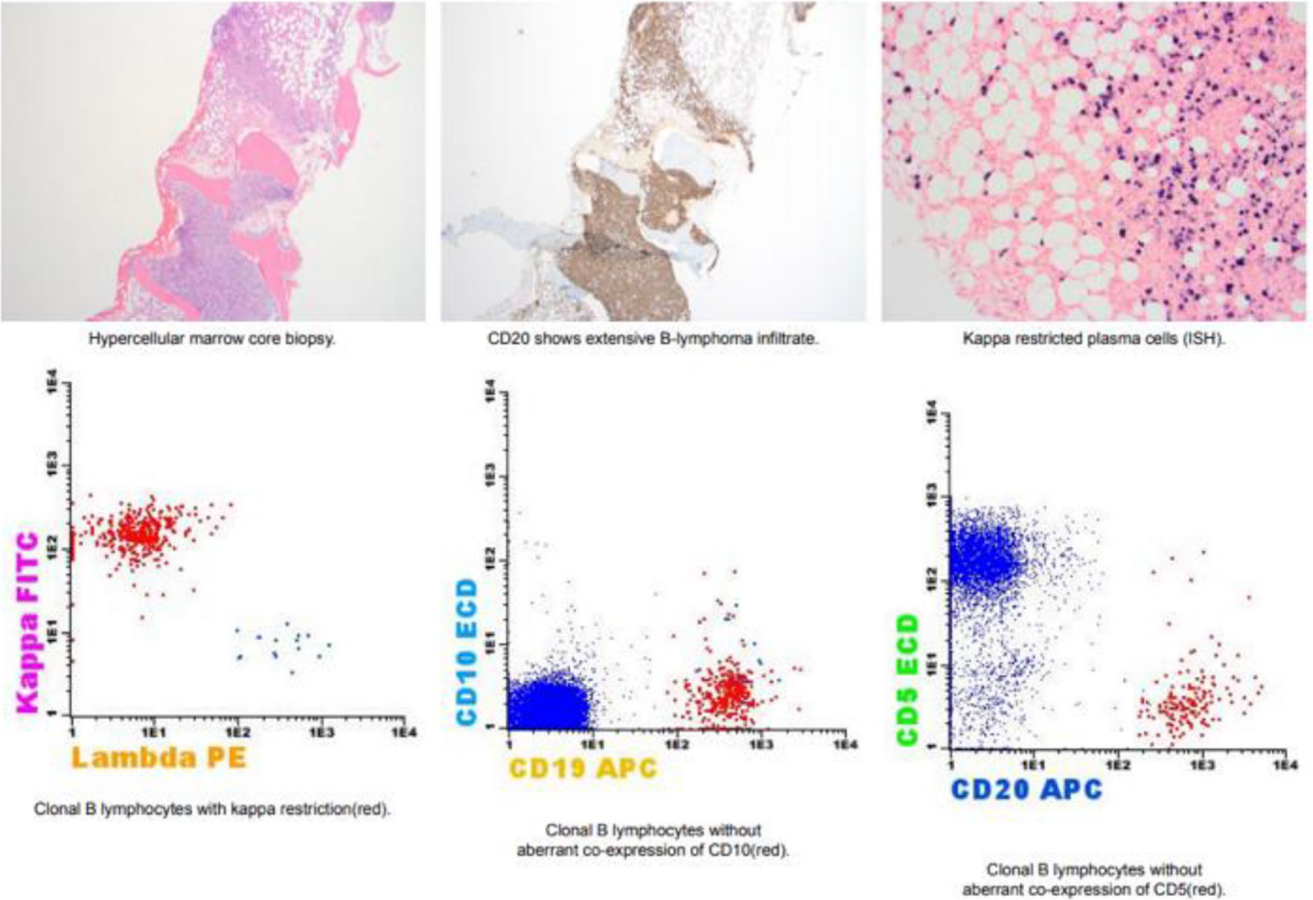
Bone marrow biopsy showing increase B-cell lymphoma marrow involvement with plasmacytic differentiation. Positive MYD88 L265P mutation

In comparison to his BM biopsy 2 years prior, the current study shows an increase in marrow involvement by B-cell lymphoma with plasmacytic differentiation. The findings in conjunction with IgM monoclonal paraprotein favor LPL, and the positive MYD88 L265P mutation analysis supports this impression. Congo red stain was negative for amyloid (Fig. 4).

The pleural effusion recurred post-thoracentesis and 420 mg ibrutinib was initiated, but due to intolerance with nausea and vomiting, required a dose reduction to 240 mg. He achieved a lasting CR as defined by National Comprehensive Cancer Network (NCCN) guidelines [4]. Seven months after initial presentation to the ED, he remains in CR and is tolerating a reduced dose of imantinib without any side effects.

## Discussion

EM pulmonary involvement in WM is uncommon and there are even fewer cases documented where a patient presented with pleural effusion prior to confirmation of WM. In addition, most malignant effusions occur after treatment for WM [1]. Our case of an untreated patient with WM/LPL and EM pleural effusion is the first documented case with pleural fluid MYD88 L265P mutation status in a community hospital setting. Our patient’s BM biopsy and IgM gammopathy from 2 years prior to his pleural effusion were concerning for LPL versus marginal zone lymphoma, but he had not manifested any other clinical, imaging, or laboratory findings at this time concerning for progression of disease to warrant treatment. In addition, the BM biopsy 2 years prior to presentation was negative for allele-specific polymerase chain reaction (AS-PCR) MYD88 L265P mutation. One study showed that BM ASPCR MYD88 L265P detection by either conventional or real-time PCR has a sensitivity and specificity of 100% and 92.1%, respectively for detection of WM [5]. Detection of MYD88 via pleural fluid genetic testing in combination with this patient’s history of IgM MGUS and past BM findings helped to guide initiating chemotherapy in this case before obtaining repeat BM biopsy.

The diagnosis of malignant pleural effusion (PF) was confirmed by PF positive MYD88 L265P mutation as well as PF flow cytometry and cytology results in a previously untreated patient with IgM MGUS and plasmacytic differentiation seen on BM biopsy. The first published case report (CR) of a patient presenting with a malignant pleural effusion attributed to WM/LPL used the techniques of standard cytology, flow cytometry, and gene rearrangement studies. The patient from this CR presented with streptococcal pneumonia bacteremia and RLL pneumonia. The malignant pleural effusion was parapneumonic but non-infectious [6].

Extramedullary pulmonary involvement in WM is uncommon. A single tertiary center reported on a longitudinal cohort of 985 patients diagnosed with WM and found pulmonary involvement in 13 patients (1.32%). In those 13 patients, the median time to the EM pulmonary presentation was six years after diagnosis and 10/13 patients had pleural effusions. Overall, 10 WM patients developed pulmonary involvement after treatment for WM and 3 had involvement prior to treatment [1].

Establishing a malignant etiology for pleural effusion in WM can be challenging, as standard techniques may be insensitive. AS-PCR for detecting MYD88 L265P mutations is more sensitive for confirming LPL/WM in pleural fluid [2]. All nine patients in this 2012 report had received at least one prior treatment regimen for WM (range 1–4). The four patients who received 480 mg of ibrutinib had complete remission. In 2015, three USA academic centers published results on 63 previously treated patients with WM enrolled in an open-label trial of 480 mg ibrutinib. They found that the presence of a positive mutation status, MYD88 (superscript) L265P, detected in 56/63 (89%) patients, correlated with favorable response to ibrutinib. Three out of 63 patients had EM pleural effusions and two thirds of patients responded, but the article does not mention their individual mutation status [2].

There are several treatment modalities for WM, as per NCCN guidelines, depending on the clinical scenario including primary, refractory, or relapsed WM. Patients with hyperviscosity syndrome may require plasmapheresis. The focus in our patient was primary treatment, and chemotherapy options here are broken down into non-stem cell toxicant medications and stem cell toxicant ones. Non-toxicant options (with some exceptions depending on dose and combination regimen) include bortezomib, rituximab, carfilzomab, cyclophosphamide, doxorubcin, thalidomide, and ibrutinib. Many of these agents can be used as mono- or combined therapy and many regimens include addition of a steroid, such as dexamethasone. Possible stem cell toxic medications include bendamustine, rituximab, caldribine, chlorambucil, fludarabine, and cyclophosphasmide. The addition of other chemotherapeutic agents, duration of therapy, or transition to different treatment regimen depend on the response to primary treatment. Possible responses include (1) complete response (2a) very good partial response (2b) partial response or (2c) minor response, and (3) no response. Criteria for a complete response include (1) normal level of IgM in your body (2) if disease presents before on imaging – no enlarged lymph nodes or organs, and (3) no symptoms or signs of WM [7].

## Conclusion

In summary, EM pulmonary involvement usually presents post-diagnosis of WM and responds well to WM-directed treatment regimens. In the setting of IgM MGUS with suspicious BM findings for WM/LPL, AS-PCR is a sensitive assay for detecting MYD88 L265P mutations in pleural fluid to support the diagnosis of malignant pleural effusion and helps guide the treatment decision to use ibrutinib. Although intolerant of ibrutinib 420 mg, our patient achieved complete and sustained remission of pleural effusion, as defined by the aforementioned NCCN guidelines, and remains in CR 7 months after initial presentation. Given early diagnosis and treatment of malignant effusion, our patient was able to avoid delay of appropriate chemotherapy and unnecessary additional thoracenteses.

## Data Availability

Data sharing not applicable to this article as no datasets were generated or analyzed during the current study.
